# What will this do to me and my brain? Ethical issues in brain-to-brain interfacing

**DOI:** 10.3389/fnsys.2015.00017

**Published:** 2015-02-25

**Authors:** Elisabeth Hildt

**Affiliations:** Center for the Study of Ethics in the Professions, Illinois Institute of TechnologyChicago, IL, USA

**Keywords:** ethics, brain-to-brain interfaces, research ethics, cross species experiments, agency, personal identity

## Abstract

Recent brain-to-brain interfacing studies provide proof of principle for the feasibility of various forms of direct information transfer between two brains, and may lead to the development of new approaches involving memory, emotions, or senses. What makes brain-to-brain interfaces unique is the transfer of information representing specific messages directly from one brain to another, without involving any activity of the peripheral nervous system or senses. The article discusses ethical issues that arise in neural interfacing. The focus is on the implications that brain-to-brain interfaces may have on the individual at the recipient side.

## Introduction

For several years now, brain-computer interfaces (BCIs) in which brain signals are used to navigate a computer, a prostheses or a technical device, have been developed in various experimental contexts (Wolpaw and Wolpaw, 2012; Grübler and Hildt, 2014). Researchers have recently taken the next step and run experiments based on connections between two brains. These so-called brain-to-brain interfaces (abbreviation: BBIs or BTBIs) involve not only a BCI component deriving information from a brain and sending it to a computer, but also a computer-brain interface (CBI) component delivering information to another brain. What results is technology-mediated brain-to-brain communication (B2B communication), i.e., direct communication between two brains that does not involve any activity of the peripheral nervous system. In what follows, ethical issues that arise in neural interfacing will be discussed after a short introduction to recent BBI experiments. In this, the focus will be on the implications BBIs may have on the individual at the CBI side of the BBI, i.e., on the recipient.

## Recent experiments

In their experiments involving a non-invasive BBI, Yoo et al. (2013) established functional links between the brain of a human volunteer and the brain of a rat. The human participant initiated an intention for intervention that was then transferred to an anesthetized rat's brain. This intention stimulated the motor area responsible for tail movement and led to involuntary tail movement. Technically, the experiment combined a BCI relying on EEG-based steady-state visual evoked potential and a CBI using transcranial focused ultrasound (FUS). FUS-based non-invasive neuromodulation of the rat brain was triggered by a computer when the human participant voluntarily started a thought process representing the intention to stimulate the rat brain. The intention was initiated by the participant looking at a strobe light flickering on a computer screen. In case of high synchronization, the FUS was triggered to transcranially modulate the anesthetized rat brain's motor cortex. Thus, the human subject was able to initiate (and thus control) the rat's tail movement via the BBI mediated “on–off” trigger.

In a similar experiment, Pais-Vieira et al. (2013) used a BBI to transfer behaviorally meaningful sensorimotor information from the brain of one rat (the “encoder” rat) to the brain of another rat (the “decoder” rat). In the study, while the encoder rat accomplished a sensorimotor task requiring the selection from two stimuli, cortical activity was recorded and transferred directly to the decoder rat's corresponding cortical areas via intracortical microstimulation. The sensorimotor information transferred via the BBI guided the decoder rat to learn similar behavioral choices, i.e., based solely on the neural patterns originating from the encoder rat. Furthermore, when the decoder animal's task performance was fed back to the encoder animal, continuous BBI operation influenced the encoder rat's neural activity and behavior. Overall, the BBI provided a “direct channel for behavioral information exchange” between two interconnected brains that allowed real-time sharing of sensorimotor information (Pais-Vieira et al., 2013, p. 6). The authors go on to state that these results “indicate that animal brain dyads or even brain networks could allow animal groups to synchronize their behaviors following neuronal-based cues.” (Pais-Vieira et al., 2013, p. 6).

A related study not based on BBIs but involving a similar donor/recipient design, was done by Deadwyler et al. (2013). Via a mathematical model they derived information encoding patterns from the hippocampus of “donor” rats well trained in a specified paradigm and delivered the information via electrical stimulation to “recipient” rats that had never before been exposed to the specific character of this task. The transfer of the model-derived hippocampal firing pattern from the trained donor animals to naïve recipient animals via stimulation facilitated the recipient animals' task performance. As there was a time delay in between the encoding phase of the task and the behavioral response in the recipient animal, the study shows the transfer of a memory code, quite in contrast to the BBI-based study by Pais-Vieira et al. (2013) which relies on the immediate induction of a motor response.

Recently, Grau et al. (2014) provided the first example of conscious transmission of information between humans via a non-invasive BBI based on a BCI using motor imagery-controlled electroencephalography and a CBI that used transcranial magnetic stimulation (TMS) to induce the conscious perception of phosphenes, i.e., the experience of seeing light. The receiver subjects on the CBI side of the BBI were able to decipher the transmitted phosphene sequences carrying encrypted messages that coded for words such as hola” or “ciao.”

Furthermore, there are speculations concerning possible future bidirectional BBI applications. For example, Yoo et al. (2013, p. 7) assume that “if both BCI and CBI are implemented between two awake human subjects, the information flow could be made bidirectional and communicative between apperceptive identities/individuals.”

Possible future applications beyond the laboratory that have been envisioned include the use of BBIs in the military or in other professional contexts where they may allow for silent commands or may serve to synchronize behavior of a larger group of individuals. Further, applications may be seen in computer gaming, in enhancing human sensory capacities or in providing support to individuals with severe motor impairments (cf. Trimper et al., 2014).

## Need for ethical reflection

The recent studies provide proof of principle for the feasibility of various forms of direct information transfer between two brains, and may lead to the development of new approaches involving memory, emotions or senses. In view of these seminal publications on BBIs allowing information transfer between animals, between humans, and between animals and humans, there is a clear need for thorough ethical reflection.

BBIs combine the recording of brain signals on the side of the sender and brain stimulation on the side of the recipient. Each of these strategies raises a broad spectrum of ethical issues that are currently being discussed in contexts such as brain-computer interfaces, deep brain stimulation, or intrasurgical brain stimulation (Freudenstein et al., 2005; Grübler and Hildt, 2014; Unterrainer and Oduncu, 2015). What makes BBIs unique, however, is the transfer of information representing specific messages directly from one brain to another, without involving any activity of the peripheral nervous system or senses. On the side of the recipient, BBIs involve a form of information input not seen so far. Furthermore, whereas the specific “meaning” of the transferred signal is clearly defined by the technical system, the behavioral implications may be far from clear.

As BBI technology currently is in the very first stages of basic research, the ethical aspects raised in this contribution are speculative to a considerable degree. Nevertheless, it is important to reflect on these issues right now. The results will be of relevance to the design of possible future BBI studies involving human subjects and will give an idea of the broader implications and possible future uses of the BBI technology.

## Some general aspects

But what exactly are the ethical issues possibly arising in brain-to-brain interfaces?

First of all, as in any kind of research involving human subjects, health-related safety issues have to be taken into consideration. In invasive systems that require surgery, there are risks concerning brain lesions. Furthermore, both in invasive and in non-invasive systems, some more indirect effects may arise: the recurring activation of specified pathways or brain regions both on the BCI part and the CBI part may influence brain functioning in various aspects.

In BBI use issues regarding agency, responsibility and liability undoubtedly will play a role (O'Brolchain and Gordijn, 2014; Vlek et al., 2014). Whereas traditional legal regulations concerning responsibility and liability in technology use may be applicable to BCIs, as suggested by several authors (Tamburrini, 2009; Clausen, 2011; Grübler, 2011), the fact that in BBIs two persons are involved complicates things considerably. The concept of “shared control” (Tamburrini, 2009), stating that in BCI use the user and the technical system together share control in achieving the output signal, undoubtedly applies to BBIs as well. Unlike in BCIs, however, there is not one person involved, but two, both of them not fully aware of their exact respective role in the system (cf. Vlek et al., 2014). Any ascription of responsibility for the outcome of any activity involving BBI functioning will be complicated by the BBI characteristic that the encoder may have initiated or significantly influenced a behavior or some sort of activity the decoder is performing (Trimper et al., 2014). Who is responsible for the consequences of activities in which BBIs are involved? The sender, i.e., the person involved in the BCI part? The recipient? The experimenter? The technical device? It may be speculated whether a concept of “hybrid agencies” (Suchman, 2007) involving several human actors might be applicable to BBIs.

Being part of a brain–brain dyad or a multi-brain system may also have complex repercussions on a person's concept of self, and raises questions concerning self-perception, individuality and body ownership (Hildt, 2011). For example, as an encoder, what is it like to be aware of another person exerting some behavior initiated or influenced by oneself? Will it be possible to clearly separate one's concept of self and the other?

Furthermore, complex problems with regard to privacy may arise, especially when the BCI component uses signals the sender is not aware of or signals the sender cannot control (Tamburrini, 2009; Trimper et al., 2014). Thus, it will be crucial to clearly define and explicitly state what kind of information will be transferred and to provide the sender with adequate measures to control the information transfer process. The same holds for the recipient in order to avoid the unconsented intrusion of information.

## Implications for the recipient

Imagine a BBI that transmits information that serves to mechanically make the decoder slightly move his left forefinger, in a method similar to the experiments run by Yoo et al. (2013) where BBI activity resulted in an anesthetized rat moving its tail. The recipient probably realizes that something is going on (his finger is moving in an automatic manner) and—being aware of the BBI and its usual function—will deduce that some information is being transferred. Thus, he probably will not ascribe authorship to himself for this movement. However, the situation will be different if the recipient is able to actively control the outcome of the information transfer, i.e., to actively control whether or not a certain movement finally occurs. For example, a person may be able to suppress the movement in question, or the BBI may solely confer a signal that serves as a stimulus for further action.

Now imagine a more flexible BBI in which various different patterns are enacted that elicit different types of reactions in the recipient. In case of five to ten different movement patterns conveyed via the BBI, would the recipient still be sure whether it is himself or the BBI that is initiating or controlling the movements? I have doubts. This uncertainty may lead him to erroneously ascribe authorship to himself for these movements (cf. Wegner, 2002; Vlek et al., 2014).

The same holds true, with even more complex implications, in cases where a BBI was able to elicit different types of emotions or memories. In a hypothetical situation where a BBI transmits a memory code that makes the recipient recall seeing a blue ball, the recipient may be clearly aware of the fact that his recurrent recalling of a blue ball may result from a BBI whose sole function is to elicit this urge. With a higher number of different stimulation patterns available, this connection will vanish so that it will be increasingly difficult for the recipient to know whether it is he or the BBI who induces a certain movement, emotion or memory. Furthermore, the transfer of emotions will considerably influence the recipient's overall well-being.

For example, in a scenario of memory transfer in humans, similar to the experiment in rats carried out by Deadwyler et al. (2013), the recipient would end up having two types of memories: his own genuine memories and the quasi-memories^1^ transferred via the BBI technology. However, he would not be able to distinguish between his own genuine memories and the quasi-memories. As for quasi-memories, the same problems arise for quasi-olfactory experiences or for quasi-emotions elicited in the context of BBI use. The recipient would no longer know for certain which of his memories, sensory experiences or emotions are genuine and which are his quasi-memories, quasi-sensations or quasi-emotions resulting from BBI information transfer. In view of this, the recipient's sense of identity would be highly questioned [cf. the philosophical debate run by Shoemaker (1970), Williams (1976), Parfit (1984), and others].

In contrast, no direct identity issues arise in the conscious information transfer described by Grau et al. (2014). In their BBI experiment, the CBI elicits phosphenes in the recipient that code for specific words. The recipient is aware of the information transfer process which involves active deciphering.

## Neural interfacing, neural grafting, and cross species experiments

It is worthwhile to compare neural interfacing with the ethical issues raised by other biomedical approaches. In particular, neural tissue transplantation is of interest here since the strategies of neural interfacing and neural grafting both involve the possibility of additional content being transferred to a brain.

Neural tissue transplantation studies in Parkinson's disease patients were run mainly in the 1980s and 1990s. They aimed at replacing loss of dopaminergic neurons in the brains of Parkinson's disease patients by transplanting mesencephalic tissue from the developing brain of aborted human embryos and fetuses into the patient's brain (Barker et al., 2013).

In the context of these clinical grafting trials, guidelines were developed that addressed ethical issues in the retrieval and use of human embryonic and fetal material. Some of them, among other aspects, contain a paragraph that serves to exclude the possibility of “personality transfer” or any risk of transfer of individual characteristics from the brain tissue donor to the recipient (British Medical Association, 1988; Dickson, 1989; Boer, 1994). For example, the “NECTAR ethical guidelines for the retrieval and use of human embryonic or fetal donor tissue for experimental and clinical neurotransplantation and research” developed by the Network of European CNS Transplantation And Restoration (NECTAR), say in point 7: “Nervous tissue may be used for transplantation as suspended cell preparations or tissue fragments” (Boer, 1994, p. 3). Allowing only cell preparations or tissue fragments to be transplanted serves to avoid the transfer of any of the donor's individual characteristics to the graft recipient.

Even if for practical reasons a “personality transfer” or the transfer of individual characteristics is very unlikely to occur in brain tissue transplantations, the guidelines considered this concern by having a paragraph that explicitly rules out this possibility in clinical transplantation studies. BBIs, however, directly involve the very issue that these guidelines attempt to avoid in the case of clinical neural tissue transplantation: the transfer of individual characteristics from a donor to a recipient, such as for example in the transfer of a memory code. Whereas in neural grafting a material substrate, i.e., brain tissue, is transferred, in BBIs there is a direct transfer of information from one brain to another. What matters from an ethical point of view is the same in both approaches. The possibility of transfer of individual characteristics. This discrepancy points to a clear need for further reflection on the ethical issues involved in the transfer of information in BBIs involving humans.

Cross species neural interfacing experiments also raise tricky ethical issues (cf. Trimper et al., 2014). With regard to research involving animals containing human material (ACHM), some ethical reflection currently is going on. For example, the government-commissioned report “Animals Containing Human Material” from the UK Academy of Medical Sciences (2011) identifies a category (Category 2) of ethically sensitive research involving ACHM which should be approached with caution but could go ahead if approval by a specialist committee on a case-by-case basis is obtained (cf. Abbott, 2011). Among others, this category includes research that involves the modification of animal brains, other than non-human primates, “that may make the brain function potentially more ‘human-like’” (Academy of Medical Sciences, 2011, p. 110).

The introduction of more “human-like” function into an animal brain via BBIs is not totally out of reach. A possible example is the BBI-induced transfer of the ability to distinguish between different words or commands (such as left, right, up, down) and to behave accordingly. Thus, some of the considerations of animals containing human material may apply to BBI technologies as well. Furthermore, in BBIs, information transfer may also emanate from an animal to a human being. For example, (Trimper et al., 2014, p. 2) speculate on possible future interspecies BBI uses such as enhancing human sensory systems or “aiding in search-and-rescue operations, linking our brains with those of the search-and-rescue animal. ” In analogy to the point raised above with regard to animals containing human material (ACHM), there is a clear need for further reflection on the ethical issues involved in attempts to modify the brains of humans in ways that might result in some “animal-like” functions.

## Conclusion

Current BBI research opens up fascinating new communication pathways but also raises considerable practical and ethical questions. One of the central questions is whether there actually is a need for direct brain-to-brain communication. At least at the moment, it seems doubtful whether there are broader applications for such a complex, rigid and expensive technology. Furthermore, in view of the complex ethical implications arising in the BBI recipient described above, the spectrum of possible ethically acceptable BBIs seems rather limited.

### Conflict of interest statement

The author declares that the research was conducted in the absence of any commercial or financial relationships that could be construed as a potential conflict of interest.

## Footnotes

^1^The term “quasi-memory” has been coined by the American philosopher Sydney Shoemaker. He describes “quasi-memory knowledge” as “a kind of knowledge of past events such that someone's having this sort of knowledge of an event does involve there being a correspondence between his present cognitive state and a past cognitive and sensory state that was of the event, but such that this correspondence, although otherwise just like that which exists in memory, does not necessarily involve that past state's having been a state of the very same person who subsequently has the knowledge.” (Shoemaker, 1970, p. 271).
